# Near-Infrared Fluorescence Imaging of Carotid Plaques in an Atherosclerotic Murine Model

**DOI:** 10.3390/biom11121753

**Published:** 2021-11-24

**Authors:** Xiaotian Wu, Amy Daniel Ulumben, Steven Long, Wataru Katagiri, Moses Q. Wilks, Hushan Yuan, Brian Cortese, Chengeng Yang, Satoshi Kashiwagi, Hak Soo Choi, Marc D. Normandin, Georges El Fakhri, Raiyan T. Zaman

**Affiliations:** 1 Gordon Center for Medical Imaging, Massachusetts General Hospital and Harvard Medical School, Boston, MA 02114, USA; amy.daniel-ulumben@compasstherapeutics.com (A.D.U.); wataru_katagiri@keio.jp (W.K.); mwilks@mgh.harvard.edu (M.Q.W.); hyuan@mgh.harvard.edu (H.Y.); cortesebd@gmail.com (B.C.); yang.cheng@husky.neu.edu (C.Y.); skashiwagi@mgh.harvard.edu (S.K.); hchoi12@mgh.harvard.edu (H.S.C.); normandin@mgh.harvard.edu (M.D.N.); elfakhri.georges@mgh.harvard.edu (G.E.F.); raiyan_zaman@yahoo.com (R.T.Z.); 2 Department of Pathology, University of California, San Francisco, CA 94143, USA; steven.long@ucsf.edu

**Keywords:** molecular-targeted fluorescent tracers, atherosclerosis, TLR4, zwitterionic, Feraheme, macrophages, NIRF

## Abstract

Successful imaging of atherosclerosis, one of the leading global causes of death, is crucial for diagnosis and intervention. Near-infrared fluorescence (NIRF) imaging has been widely adopted along with multimodal/hybrid imaging systems for plaque detection. We evaluate two macrophage-targeting fluorescent tracers for NIRF imaging (TLR4-ZW800-1C and Feraheme-Alexa Fluor 750) in an atherosclerotic murine cohort, where the left carotid artery (LCA) is ligated to cause stenosis, and the right carotid artery (RCA) is used as a control. Imaging performed on dissected tissues revealed that both tracers had high uptake in the diseased vessel compared to the control, which was readily visible even at short exposure times. In addition, ZW800-1C’s renal clearance ability and Feraheme’s FDA approval puts these two tracers in line with other NIRF tracers such as ICG. Continued investigation with these tracers using intravascular NIRF imaging and larger animal models is warranted for clinical translation.

## 1. Introduction

Cardiovascular disease remains the largest cause of mortality in the United States, accounting for about 860,000 deaths in 2017 [1]. Of these deaths, 42.6% were attributed to coronary heart disease specifically, paving the way for imaging research to detect the underlying atherosclerotic plaques for early diagnosis and intervention [1,2]. Successful imaging of vulnerable plaques involves determining their morphology and composition, specifically, the detection of an inflamed, thin-cap fibroatheroma (TCFA) featuring a lipid-rich, atheromatous core, a thin fibrous cap with macrophage and lymphocyte infiltration, decreased smooth muscle cell content, and expansive arterial remodeling [3].

The difficulty of detecting specific plaque components with one single technology has resulted in the development of multimodal/hybrid intravascular imaging [4]. The potential complementary technologies involve imaging techniques such as angioscopy, intravascular optical coherence tomography (IVOCT), ultrasound (IVUS), near-infrared spectroscopy (NIRS), near-infrared fluorescence (NIRF), fluorescence lifetime imaging (FLIm), and photoacoustic imaging (IVPA).

Imaging of the blood vessel is well suited for optical imaging as the background signal from autofluorescence is relatively low, resulting in potentially large signal sensitivity. Furthermore, the targeting of important metabolic processes can be achieved through fluorescence energy transfer or bioluminescence [5]. Visualizing plaque macrophages through an NIRF probe targeting inflammatory molecular pathways has been proposed as a promising approach [6]. Indocyanine green (ICG) is one such NIRF dye that is Food and Drug Administration (FDA)-approved and had its first-in-human investigation for atheroma targeting in 2016 [7,8]. ICG signals were detected in human plaques but were low in vivo since ICG has a short half-life in blood and is readily taken up by the liver [7,8]. Ikeda et al. used iron oxide nanoparticles conjugated with ICG to accumulate in macrophages to obtain stronger NIRF signals in vivo, but observed a significant accumulation of the dye in the liver, followed by the spleen [9]. In this paper, we investigate two macrophage-targeting fluorescent tracers, TLR4-ZW800-1C and Feraheme-Alexa Fluor 750, with the following properties to improve NIRF imaging specifically for atherosclerotic plaque detection.

TLR4-ZW800-1C targets toll-like receptor 4 (TLR4), which plays critical roles in macrophage differentiation into foam cells and inflammation and has been recognized as an important biomarker for atheroma development and atherosclerotic plaque instability [10,11]. ZW800-1C is a zwitterionic near-infrared (NIR) fluorophore that exhibits low serum binding, ultralow nonspecific tissue background, and rapid elimination from the body via renal filtration in its unconjugated form [12]. In a previous rodent study by Hyun et al., ZW800-1C was shown to be rapidly cleared through kidney filtration with nearly negligible liver uptake, and the elevated kidney signals decreased significantly in accordance with urinary excretion over 4 h [12]. Serum proteins prevent the dimerization of ZW800-1C, resulting in the lack of nonspecific tissue/organ uptake in vivo due to the microaggregations [12].

Today, atherosclerotic plaques are still difficult to detect due to their small size, motion, and obscured signal from adjacent nonspecific tissues. Therefore, the ultralow nonspecific tissue background of ZW800-1C is an extremely desirable feature for improving the detection of unstable atherosclerotic plaque due to its high signal-to-noise ratio (SNR) compared to its surrounding tissue. Another important advantage of ZW800-1C in atherosclerotic plaque imaging as a fluorescent agent is its high renal clearance, as opposed to FDA-approved ICG’s high uptake in the liver. In our previous work, an anti-TLR4 antibody conjugated to ZW800-1C (TLR4- ZW800-1C) has been successful in detecting tumor-associated macrophages (TAMs) in liver cancer in a mouse model [13]. In this work, we propose to use NIRF imaging with TLR4-ZW800-1C, targeting macrophage TLR4 in this novel application to locate inflamed plaques.

Feraheme-Alexa Fluor 750 consists of Feraheme (ferumoxytol), an iron oxide nanoparticle that has FDA approval for the treatment of iron anemia [14,15]. The molecule is recognized by proinflammatory and anti-inflammatory macrophages via scavenger receptor type AI/II and has been extensively used off label as a Magnetic Resonance Imaging (MRI) contrast agent for these cell types in various applications, including carotid atherosclerosis [15,16]. Previous work has also shown that these nanoparticles can be modified for fluorescent labeling and imaging of these cell types [17]. Here, the Feraheme (FH) is conjugated with Alexa Fluor 750 (AF750), a commercial NIRF dye for research purposes that features pH insensitivity over a wide molar range (Thermo Fisher Scientific, Waltham, MA, USA). Feraheme’s FDA approval and previous studies showing its successful application in atherosclerosis detection in MRI imaging prompt us to investigate its utility in NIRF imaging as well, specifically for intravascular imaging for the detection of coronary artery disease and the evaluation of the arterial wall before any interventions.

In this paper, we hypothesize that both TLR4-ZW800-1C and FH-AF750 can allow us to locate inflamed plaques with NIRF imaging through their distinct macrophage targeting mechanisms.

## 2. Materials and Methods

### 2.1. Tracers Synthesis

#### 2.1.1. TLR4-ZW800-1C

A *N*-Hydroxysuccinimide (NHS) ester form of ZW800-1C was synthesized and conjugated with a TLR4/MD-2 complex monoclonal antibody (MTS510, Thermo Fisher Scientific, Waltham, MA, USA), as previously described by Hyun et al. [12]. Briefly, 10 equivalents of ZW800-1C NHS ester was added to an anti-TLR4 antibody in PBS, pH 8.0, and incubated at room temperature for 3 h with continuous stirring. The reaction mixture was then purified using a mini Bio-Gel P-6 desalting column (Bio-Rad, Hercules, CA, USA) three times with saline and concentrated using a 10K molecular-weight cutoff (MWCO) spin column (Vivaspin 500, MWCO = 10 kDa). The labeling ratio between the anti-TLR4 antibody and ZW800-1C was 1.40, calculated based on the Beer–Lambert law by determining the concentration of each compound. TLR4-ZW800-1C was analyzed for optical properties using a UV–Vis–NIR spectrophotometer (USB-ISS-UV/VIS, Ocean Optics, Dunedin, FL, USA).

#### 2.1.2. Feraheme-Alexa Fluor 750 (FH-AF750)

FH-AF750 was synthesized using previous published methods [17]. Briefly, washed and purified Feraheme was incubated with hydroxybenzotriazole (HOBT) and N-(3-dimethyaminopropyl)-N′-ethylcarbodiimide (EDC) in MES buffer (pH 6.0–6.4) at room temperature for 20 min. Ethylenediamine (1 M) was added to the mixture and incubated at 50 °C for 90 min. This raw FH–amine solution was then purified on a Sephadex G-25 column, and concentrated using 50 kDa Amicon centrifugation filters. This purified FH–amine compound then underwent conjugation with an AF750-NHS ester (Thermo Fisher Scientific, Waltham, MA, USA). The final product, FH-AF750, was again purified in a Sephadex G-25 column and reconcentrated by 50 kDa Amicon centrifugation filters. Nanoparticle (NP) concentration, and the payloads of AF750/NP of the final product, were determined spectrophotometrically.

### 2.2. Animal Model Development

All animal procedures were conducted according to Massachusetts General Brigham (MGB) IACUC-approved protocol 2019N000104. A carotid atherosclerotic model was developed using 8-week-old, male FVB/NJ mice (The Jackson Laboratory, Bar Harbor, ME, USA). Mice (*n* = 14) were kept on a 0.2% high-cholesterol diet (HCD, TD.88137, Envigo, Hackensack, NJ, USA) for a total of eight weeks after initial acclimation. After four weeks on HCD, the mice were intraperitoneally (IP) injected with Streptozotocin (STZ, 40 mg/kg, Sigma-Aldrich, St. Louis, MO, USA) for 5 consecutive days to induce diabetes. After 5 injections, 3 more STZ doses were injected based on blood glucose test results. All mice were diabetic after 8 STZ injections (8 h fasting blood glucose > 119 mg/dL, mean ± SD = 164 ± 35 mg/dL). Mice weights were closely monitored throughout the process in order to track their wellbeing. Two weeks after the diabetic induction, the left carotid artery (LCA) was surgically ligated with a silk suture (6-0 Perma-hand, Ethicon, Somerville, NJ, USA) to mimic carotid stenosis by accelerating plaque development, and the right carotid artery (RCA) was kept intact as a negative control.

### 2.3. Imaging Protocol

At the end of the eight weeks, the mice were fasted for 6 h before TLR4-ZW800-1C (*n* = 8, mice group T1–8, 10 nmol) or FH-AF750 (*n* = 6, mice group F1–6, 5 mg/kg) was injected retro-orbitally for imaging. The mice were imaged in vivo with an in-house-built multispectral NIRF imaging system (see Supplementary Figure S1, Excitation: 760 nm, Emission: 785 nm long pass) at various time points (baseline (0 h), 1 h, 56.5 h for T1–8 only, 7–8 h for F1–6 only, and 24 h for F1–6 only) [13]. Baseline images were acquired before the contrast agent injection. Food was replenished after the 1 h imaging time point. Mice were euthanized at the final imaging time point (6.5 h for the T1–8 group and 24 h for the F1–6 group).

Organs were harvested after a blood flush (3 mL normal saline through the left ventricle) for ex vivo NIRF imaging and histological analyses. The specific organs of interest were the LCA and RCA, harvested together with the aortic arch and the heart. To study biodistribution of the tracers, ex vivo NIRF imaging was also performed on the harvested heart, lung, liver, pancreas, spleen, kidney, duodenum, intestine, and muscle tissues.

### 2.4. Data Analysis

The intensity images were saved as 16-bit depth, 512-by-512.png files. Image analysis of the NIRF imaging was performed with custom-developed MATLAB tools, and signal-to-background ratios (SBRs) were calculated based on image intensities over regions of interests (ROIs) and background. Similarly, for images using muscle as references, the signal-to-muscle ratio (SMR) was calculated as follows:

(1) $$\mathrm{SMR}\;=\frac{I_{\mathrm{ROI}}}{I_{\mathrm{Auto}}},$$

where $I_{\mathrm{ROI}}$ denotes the average intensity of an ROI and $I_{\mathrm{Auto}}$ represents the intensity of the muscle [13].

For in vivo imaging, ROIs were defined over a trapezoidal region on the chest of the mice in a supine position (ventral side up). For LCA and RCA, ROIs were defined by the maximal intensity regions in the LCA and a corresponding region in the RCA. For other organs, the ROIs were defined over the entirety of the harvested organs.

SBR, SMR, and other ratios were presented as means with standard deviations (SDs), and standard errors (SEs). Paired t-tests were conducted for LCA and RCA comparison (α = 0.05). Pearson correlation coefficients were computed for the intravital imaging of both dyes and for signal vs. thickness ratios of both T and F mice cohorts. A one-way ANOVA was conducted for biodistribution analysis. All computations were performed using MATLAB (MathWorks, Natick, MA, USA) and Microsoft Excel (Microsoft, Redmond, WA, USA).

### 2.5. Histological Protocol

The LCA and RCA were collected after ex vivo imaging for histological analysis. Samples were fixed in 10% formalin, then embedded in paraffin and sectioned. Hematoxylin and eosin (H&E), Trichrome, and CD68 stains were acquired for the LCA and RCA paraffin slides for histological analysis by a pathologist.

## 3. Results

**Figure 1:**
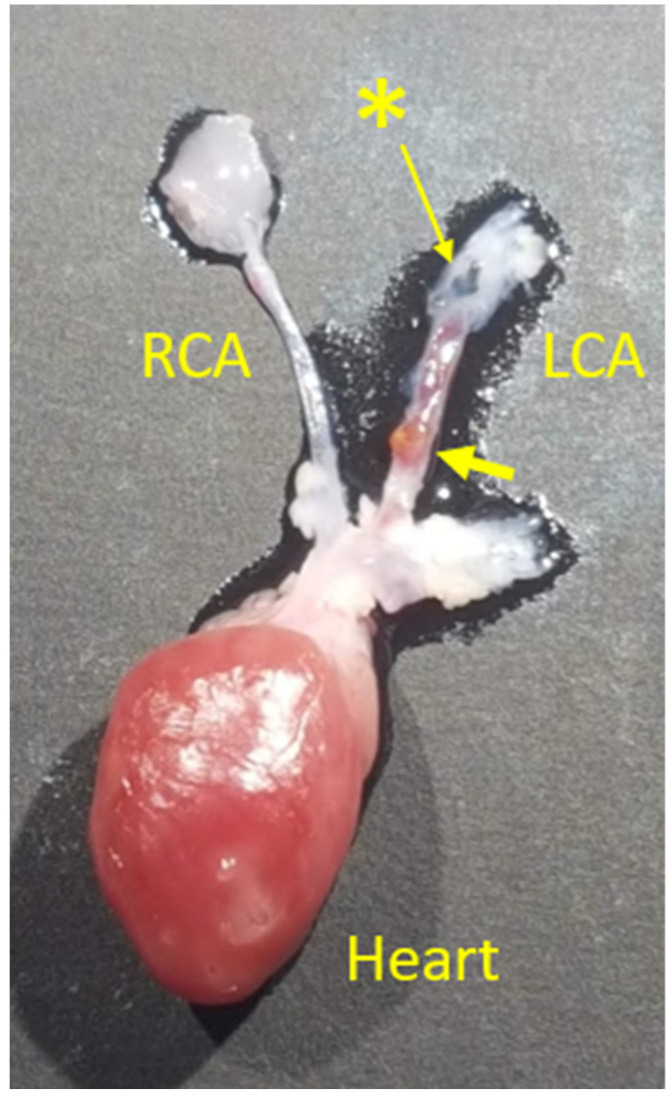
Significant plaque formation (large yellow arrow) in the left carotid artery (LCA) of F1. Asterisk (*) marks the ligation region with a black silk suture on the LCA. The LCA is visibly thicker than the control right carotid artery (RCA) due to atherosclerotic plaques and macrophage infiltration.

Of the 14 mice that completed the 8-week protocol, eight mice were in the TLR4-ZW800-1C group (T1–8) and six were in the FH-AF750 group (F1–6). Two (T1 and T2) in the TLR4-ZW800-1C group did not show visible signs of plaque build-up in the LCA or exhibit a thickening of the LCA proximal to the ligation. A white light image of a harvested heart with plaque formation in the LCA and control RCA is shown (Figure 1). The LCAs of mice with plaque (T3–8, F1–6) were visibly thicker than the RCA due to the stenosis caused by the ligation, with a combined mean of a 38% increase in thickness (Table 1).

**Figure 2:**
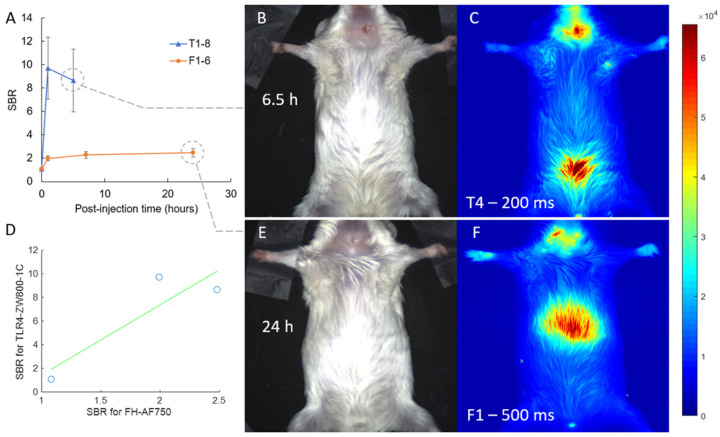
Intravital NIRF imaging. (**A**) SBR values for chest region including incision site at various imaging time points for both T and F cohorts. (**B**,**E**) Color image of mice in supine position. (**C**,**F**) Corresponding 785 nm fluorescence image (at 200 and 500 ms exposure to exaggerate contrast) showing high signal from the chest region. TLR4-ZW800-1C (Group T1–8) show high signal in the bladder at endpoint (**C**), whereas FH-AF750 show high uptake in the liver (**F**). (**D**) SBR correlation between both dyes (r = 0.89, *p* = 0.3).

We conducted intravital NIRF imaging of these mice at various time points after injecting each mouse cohort with their respective tracers (Figure 2). Baseline (pre-injection) in vivo images over the chest region showed no significant fluorescence signal for all mice, with an average SBR ± SD of 1.08 ± 0.01 at 100 ms exposure and of 1.71 ± 0.08 even at 1000 ms exposure. All SBR values henceforth are reported at the short 100 ms exposure for standardization to avoid oversaturated pixels. In vivo imaging of the chest region at the 1 h time point resulted in SBR ± SD of 9.72 ± 7.49 for T1–8 and 1.99 ± 0.47 for F1–6. Right before euthanasia, T1–6 had chest SBR values of 8.66 ± 7.57 at the 5–6.5 h time point. F1–6 had chest SBR values of 2.48 ± 0.93 at the 24 h euthanasia time point. Correlation between both dyes for 0 h, 1 h, and pre-euthanasia endpoints (6.5 h for T1–8, 24 h for F1–6) gave a Pearson’s r value of 0.89 (*p* = 0.30). Both dyes exhibited high fluorescent signal over the chest region via in vivo imaging, but specific signals from individual carotid arteries could not be distinguished, possibly due to low signal and high scatter through the skin. This heightened signal could also stem from inflammation at or near the incision site; hence, ex vivo images were taken to evaluate signals stemming from the LCA and RCA only.

**Figure 3:**
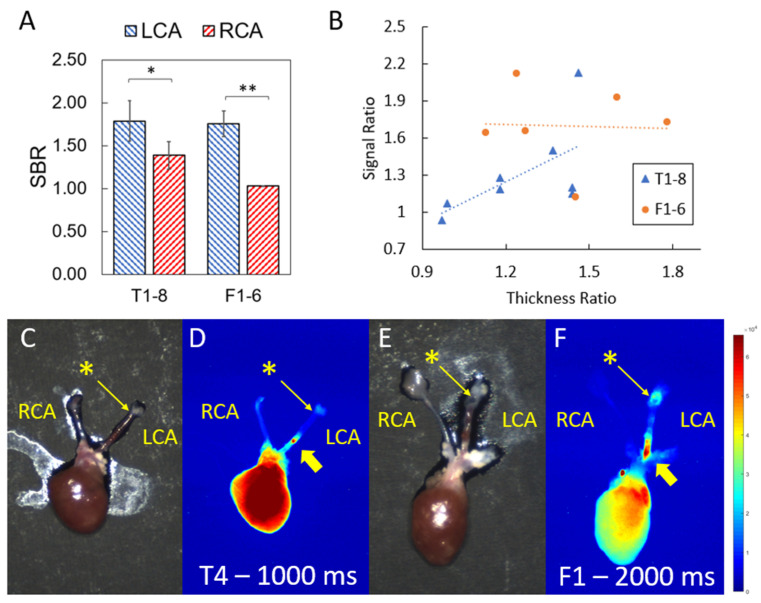
**(A**) Signal-to-background ratio (SBR mean with SE) of left vs. right carotid arteries (LCA vs. RCA) of mice tagged with TLR4-ZW800-1C (T1–8) and with FH-AF750 (F1–6) at 100 ms exposure time. Paired t-tests with * *p* < 0.05, ** *p* < 0.01. (**B**) Signal and thickness (LCA vs. RCA) ratio correlation for T1–8 (r = 0.61, *p* = 0.11) and F1–6 (r = −0.04, *p* = 0.93). (**C**,**E**): Color image of ex vivo mice heart (mouse T4 and F1) with aortic arch and carotid arteries. Asterisk (*) marks the ligation region on the left carotid artery (LCA). Thick yellow arrows point to the high concentration of fluorescent signal from the plaque region in the LCA. (**D**,**F**) Corresponding 785 nm fluorescence image (at 1000 and 2000 ms exposure to exaggerate contrast of the carotid arteries) showing the visible plaque region tagged with TLR4-ZW800-1C and FH-AF750, respectively.

After euthanasia and harvest, SBR fluorescence signals in the LCA vs. RCA were computed and are shown in Table 2 and Figure 3 at 100 ms exposure time. With either dye, the LCA exhibited significantly higher signals than the control RCA for mice with visible LCA thickening and signs of plaque development, a mean 31% higher signal for T1–8 and 70% for F1–6. Excluding the two mice (T1–2) that did not show visible LCA thickening, the mean increase in the LCA signal over the RCA for T3–8 was 40%. Correlations between the signal ratios and thickness ratios give r = 0.61, *p* = 0.11, r = −0.04, and *p* = 0.93 for T1–8 and F1–6. Color/NIRF images of the harvested heart, aortic arch, LCA, and RCA, and of the other harvested organs, are shown in Figure 3C–F. The region of heightened signal in the LCA seems to be more concentrated on a region more proximal to the heart than to the ligation site. The two mice that did not exhibit physical signs of pathology (T1 and T2) evidently had an LCA-to-RCA ratio of 0.94 and 1.07, respectively. The mean SBR in the LCA for all eight mice in the TLR4-ZW800-1C group was 1.79 ± 0.66, and 1.39 ± 0.45 for the control RCA. The mean SBR in the LCA for all six mice in the FH-AF750 group was 1.75 ± 0.37, and 1.03 ± 0.01 for the control RCA.

**Figure 4:**
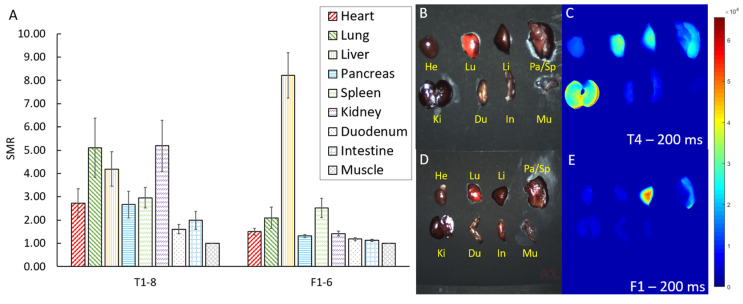
(**A**) Signal-to-muscle ratios (SMR with SE) of various organs of mice tagged with TLR4-ZW800-1C (Group T1–8, 6.5 h harvest) and with FH-AF750 (Group F1–6, 24 h harvest) at 100 ms exposure time. For T1–8, uptake is highest in the kidney, lung, and liver. For F1–6, uptake is highest predominantly in the liver, followed by the spleen. (**B**,**D**) Color image of ex vivo mice organs. (**C**,**E**) Corresponding 785 nm fluorescence image (at 200 ms exposure) showing predominant uptake of TLR4-ZW800-1C in kidneys, lungs, and liver and of FH-AF750 in the liver and spleen. Organs were coded as follows: He is the heart, Lu is the lungs, Li is the liver, Pa/Sp is the pancreas/spleen, Ki is the kidney, Du is the duodenum, In is the intestine, and Mu is the muscle.

Biodistribution of both contrast agents within the heart, lung, liver, pancreas, spleen, kidney, duodenum, intestine, and muscle were analyzed in ex vivo imaging and are shown in Table 3 and Figure 4. For T1–8, the highest signal-to-muscle ratios (SMR mean ± SD) come from the kidney (5.19 ± 3.12), lung (5.11 ± 3.62), and liver (4.19 ± 2.08). For F1–6, uptake is predominantly in the liver (8.22 ± 2.38), followed by in the spleen (2.52 ± 1.00). For both agents, muscle tissue exhibited the lowest fluorescent signal and thus served as a reference. These biodistribution results are consistent with known probe uptake and excretion routes in the literature [13,17]. There is a statistically significant difference in biodistribution of the dye using muscle as reference (for T1–8, ANOVA: F = 3.356, df = 7, *p* < 0.01, and for F1–6, ANOVA: F = 33.432, df = 7, *p* < 0.001).

In accordance with literature, although FH-AF750 was heavily taken up by the liver, TLR4-ZW800-1C’s absorption into the lungs and into the kidneys for clearance presents better clinical viability [13,18,19,20]. Both dyes exhibited relatively high uptake in the heart compared to the carotid arteries. The high uptake in the heart for TLR4-ZW800-1C was considered to reflect the physiological expression of TLR4 in cardiomyocytes [21]. Although heightened signal in the heart may pose an issue for plaque detection in the coronary arteries, especially due to their proximity, applications of these dyes in intravascular imaging may be more successful than noninvasive whole-body imaging.

**Figure 5:**
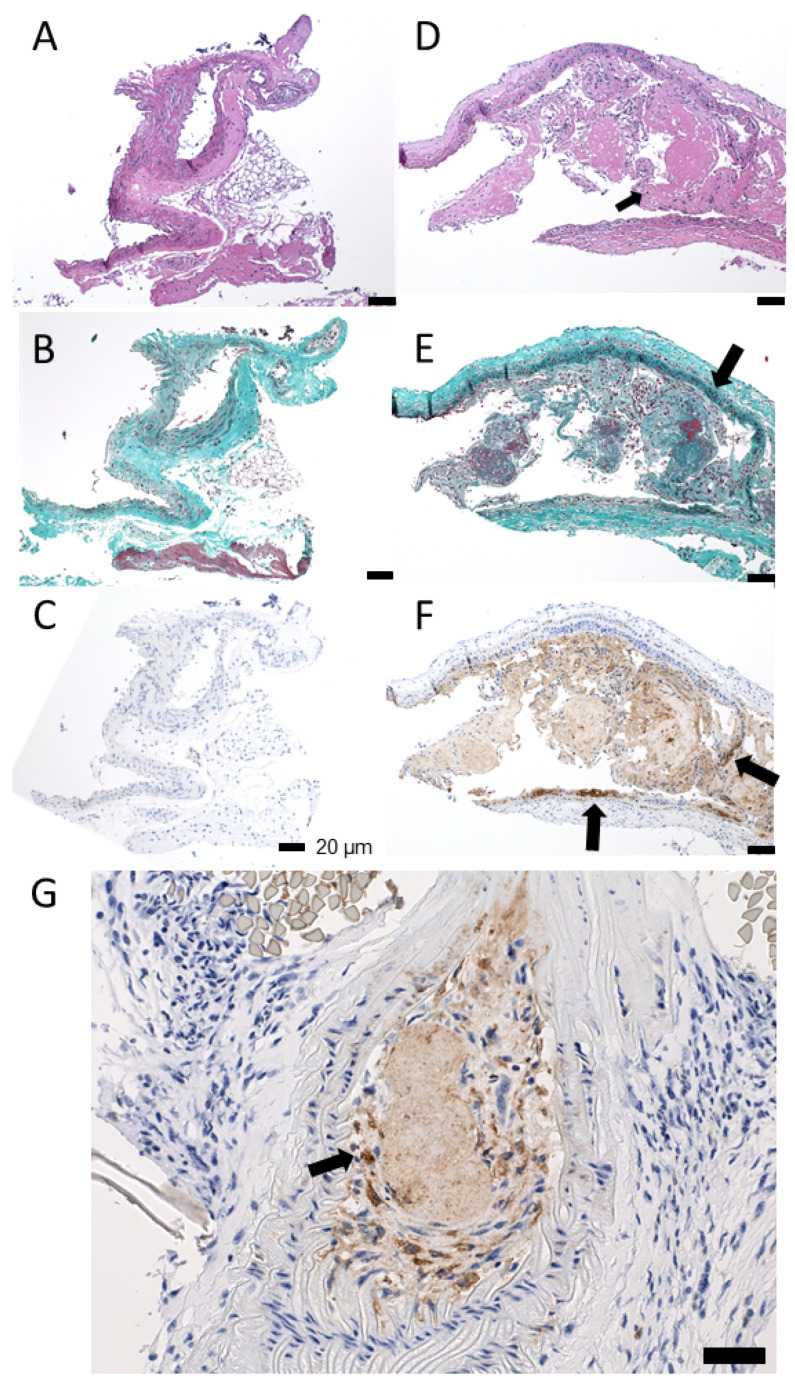
Histology of carotid samples: (**A**–**C**) show normal/control sections of the RCA vessels at 100x without significant pathology, whereas (**D**–**F**) show diseased LCA samples. (**A**) An H&E cross section without luminal thrombus formation or an abnormal infiltrate of lymphocytes, histiocytes, or collagen in the wall or lumen of the vessel. (**B**,**C**) Same segment of vessel without increased green staining of collagen (**B**) or increased brown cytoplasmic staining, which would indicate macrophage infiltration (**C**). (**D**) H&E, 100×. Cross section of abnormal vessel shows luminal dilation, filled with plaque including large zones of acellular/degenerated bright pink fibrinous material, mixed with cellular aggregates of material including macrophages, chronic inflammatory cells and lymphocytes, and collagen. The wall of the vessel is also abnormal and infiltrated by chronic inflammatory cells, including lymphocytes and macrophages. (**E**) Trichrome, 100×. Dilated abnormal cross section of vessel shows increased collagen deposition (highlighted by increased green staining) in both the luminal thrombus and in the wall of the artery itself. Notice the increased collagen/green staining of the wall of the vessel in the upper and right-hand side of the photo vs. the lack of increased staining in the wall of the vessel seen in the lower left segment of this vessel. (**F**) 100× and (**G**) 200×. CD68 (macrophage stain) highlights an infiltration of macrophages into the wall of the vessel. The immunoperoxidase stain deposits brown staining pigment in the cytoplasm of the histiocytes in this image. Scale bars are 20 µm.

Finally, the LCA and RCA were collected for histology verification. Histology slides show clear differences between the control RCA and the diseased LCA (Figure 5). The control RCA presents as a normal vessel, suggesting that atherosclerosis was not systemically developed in this model, whereas the diseased LCAs show atypical cell proliferation and accumulation of macrophages and lymphocytes via H&E, Trichrome, and CD68 stains.

## 4. Discussion

In summary, NIRF imaging in conjunction with a single intravenous injection of TLR4- ZW800-1C or FH-AF750 was able to detect atherosclerotic plaques in the LCA with high sensitivity, as compared to the control RCA. T1–8 showed a 31% increase and F1–6 showed a 70% increase in signal in the diseased LCA over the RCA control even at a short 100 ms exposure time. Since TLR4 is involved in critical steps of plaque formation, and Feraheme is readily taken up by macrophages involved in the resulting inflammatory process, both fluorescent tracers show promise in being able to identify high-risk plaques likely to progress and can be included in the arsenal of molecular probes for NIRF imaging of atherosclerosis [7]. The results of this study are encouraging even with small sample sizes (T1–8 and F1–6), since each animal served as its own control.

A limitation of the study is that our NIRF imaging system is suited for whole-body rodent imaging, and we have not thoroughly investigated tuning the system for optimal noninvasive imaging. Additionally, NIRF imaging suffers from scatter through deep tissue, which makes noninvasive coronary artery imaging difficult. NIR-II imaging with deeper penetration into soft tissue, reduced scattering, and lower auto-fluorescence may be promising for this task [22,23]. Another limitation of the study is the histological validation. Although we histologically verified the presence of disease in the left vs. right carotid vessels, further histological analysis is warranted to verify specificity and colocalization of the NIRF probes.

Future investigation will be required to determine the efficacy of more localized noninvasive imaging for the improved sensitivity and specificity of plaque detection in the arteries. Clinical translation of this technology would likely involve intravascular NIRF imaging, as localization of these early plaques is crucial for intervention, and fluorescent scatter through tissue would mitigate the high sensitivity signal. Continued investigation in larger animals using intravascular NIRF catheters is the next step. In addition, investigation of other antibodies conjugating with tracers is important for more specific targeting of inflammatory components that may better characterize disease stage or severity.

## Figures and Tables

**Table 1:** Ratio of LCA to RCA thickness.

	T1	T2	T3	T4	T5	T6	T7	T8	Mean	SD	SE
LCA:RCA Thickness Ratio	0.97	0.99	1.37	1.46	1.44	1.18	1.18	1.44	1.25	0.20	0.07
			F1	F2	F3	F4	F5	F6			
			1.60	1.27	1.45	1.13	1.78	1.24	1.41	0.25	0.10

T1–2 did not show visible signs of LCA thickening. T3–8 and A1–6 exhibited an average 38% increase in thickness of the LCA as compared to the RCA control. SD, standard deviation; SE, standard error.

**Table 2:** Fluorescent signal ratios between left and right carotid arteries (LCA:RCA).

	T1	T2	T3	T4	T5	T6	T7	T8	Mean	SD	SE
LCA:RCA Ratio	0.94	1.07	1.50	2.13	1.15	1.19	1.28	1.20	1.31	0.37	0.13
			F1	F2	F3	F4	F5	F6			
			1.93	1.66	1.12	1.64	1.73	2.12	1.70	0.34	0.13

Mice were tagged with TLR4-ZW800-1C (Group T1–8, 6.5 h harvest) and with FH-AF750 (Group F1–6, 24 h harvest) and imaged with 100 ms exposure time. With the exception of T1–2, whose LCA did not exhibit visible signs of pathology, signal in the LCA is significantly higher than in the RCA for the rest of the mice using either dye (T3–8, F1–6).

**Table 3:** Fluorescent biodistributions of TLR4-ZW800-1C and FH-AF750.

Signal-to-Muscle Ratio (SMR) ± SD									
	Heart	Lung	Liver	Pancreas	Spleen	Kidney	Duodenum	Intestine	Muscle (Reference)
T1–8	2.72	5.11	4.19	2.66	2.95	5.19	1.60	1.99	1.00
	±1.74	±3.62	±2.08	±1.61	±1.24	±3.12	±0.55	±1.09	±0.00
F1–6	1.50	2.10	8.22	1.31	2.52	1.42	1.19	1.13	1.00
	±0.34	±1.13	±2.38	±0.15	±1.00	±0.25	±0.15	±0.11	±0.00

Signal ratios (±SD) of various organs to muscle tissue of mice tagged with TLR4-ZW800-1C (Group T1–8, 6.5 h harvest) and with FH-AF750 (Group F1–6, 24 h harvest) at 100 ms exposure time.

## Data Availability

Data are presented in this manuscript. Additional images are available upon request from the authors.

## Supplementary Materials

The following are available online at https://www.mdpi.com/article/10.3390/biom11121753/s1, Figure S1: NIRF imaging system.
