# Supplementary material for: Near-Infrared Fluorescence Imaging of Carotid Plaques in an Atherosclerotic Murine Model
Source: Biomolecules. 2021 Nov 24;11(12):1753. doi: 10.3390/biom11121753 (PMC8698491; doi:10.3390/biom11121753)
Supplement: Supplementary file 1 [file biomolecules-11-01753-s001.zip › biomolecules-1447651-supplementary.pdf]

Supplementary Materials for Biomolecules-Special Issue submission
"Near-infrared fluorescence imaging of carotid plaques in a atherosclerotic murine model" X.Wu et al.

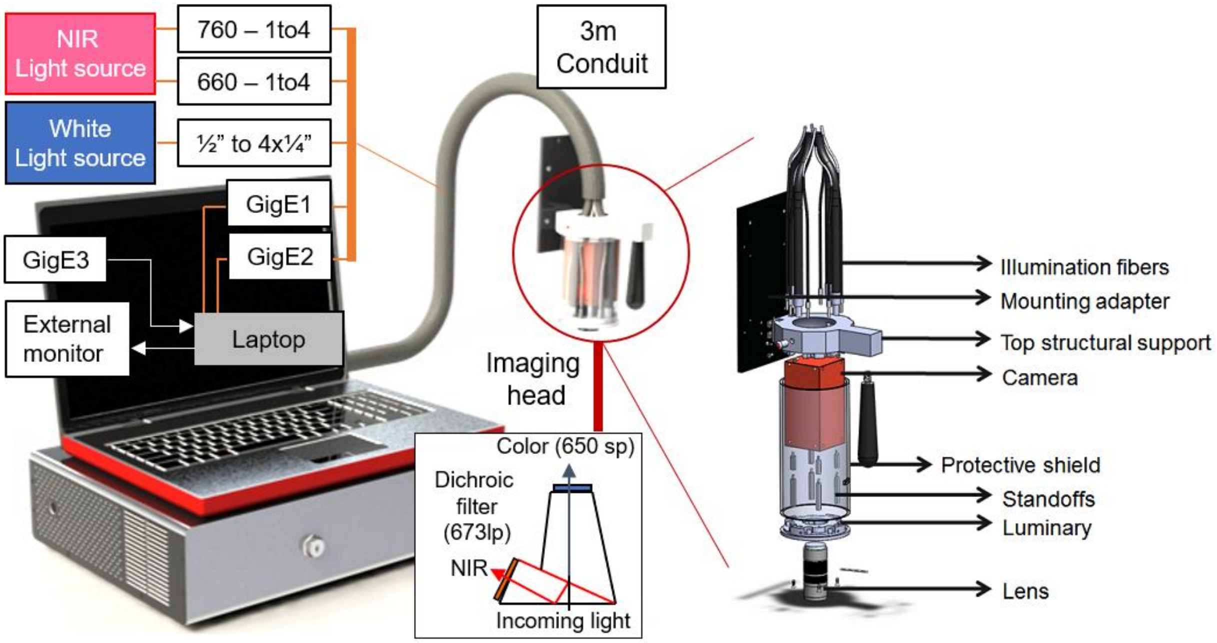

Figure S1: NIRF imaging system [9] used that has white light and NIR sources (660 and 760 nm). The 760 nm excitation source was used for this study.
